# Responses of arbuscular mycorrhizal wheat to salinity: from symbiotic signaling to stress adaptation

**DOI:** 10.1080/15592324.2026.2733240

**Published:** 2026-09-23

**Authors:** Hakim Zamir, Fawad Rauf, Zameer Hussain Jamali, Shahrukh Khan, Usman Zulfiqar, Mohammed S. Alotaibi, Muydinjon Muminov, Sokhibjon Abdusamatov, Mayank Anand Gururani, Shakal Khan Korai

**Affiliations:** a Joint International Research Laboratory of Agriculture and Agri-Product Safety, The Ministry of Education of China, Institutes of Agricultural Science and Technology Development, Yangzhou University, Yangzhou, Jiangsu, People's Republic of China; b College of Bioscience and Biotechnology, Yangzhou University, Yangzhou, People's Republic of China; c Department of Applied Ecology, Faculty of Agriculture and Technology, University of South Bohemia in Ceske Budejovice, Ceske Budejovice, Czech Republic; d College of Animal Science and Technology, Yangzhou University, Yangzhou, People's Republic of China; e Department of Agronomy, Faculty of Agriculture and Environment, The Islamia University of Bahawalpur, Bahawalpur, Pakistan; f Department of Biology, Nakhchivan State University, Nakhchivan, Azerbaijan; g Department of Biology, Tarabah University College, Taif University, Taif, Saudi Arabia; h Department of Chemistry, Andijan State University, Andijan, Uzbekistan; i Department of Startup Projects, Kokand University, Kokand City, Uzbekistan; j Department of Microbiology and Biotechnology, National University of Uzbekistan, Tashkent, Uzbekistan; k Department of Biology, College of Science, United Arab Emirates University, Al Ain, United Arab Emirates

**Keywords:** Arbuscular mycorrhizal symbiosis, wheat, salinity, ion homeostasis, redox signaling, hormonal crosstalk, arbuscule functionality

## Abstract

Salinity is a major constraint to wheat productivity, imposing osmotic stress, ionic imbalance, and oxidative pressure that compromise growth and yield. Arbuscular mycorrhizal fungi can improve wheat performance under salinity through effects extending beyond nutrient acquisition. This review examines the context-dependent symbiosis between wheat and arbuscular mycorrhizal fungi, in which reciprocal signaling and resource exchange influence stress adaptation. Direct studies, particularly in durum and bread wheat, indicate that AM colonization can improve K⁺/Na⁺ balance, nutrient acquisition, water relations, membrane stability, antioxidant regulation, osmoprotectant metabolism, and stress-responsive gene expression. By contrast, detailed mechanisms of presymbiotic communication, fungal-signal perception, nuclear Ca^2+^ decoding, transcriptional accommodation, arbuscule development, and plant-to-fungus lipid transfer have been characterized mainly in AM model plants and other cereals. These conserved pathways provide a mechanistic framework for interpreting, rather than presuming, their operation in salt-stressed wheat. Integrating these evidence levels indicates that AM-associated benefits arise from coordination among ion and water transport, reactive oxygen species and redox regulation, hormonal crosstalk, carbon allocation, and arbuscule-mediated nutrient exchange rather than from enhancement of a single protective trait. The magnitude and nature of these benefits are context-dependent, influenced by wheat genotype, fungal identity, salinity intensity, nutrient status, and carbon cost-benefit trade-offs. We identify priorities for wheat-specific functional validation, spatial and temporal analysis of Ca^2+^, reactive oxygen species, hormones, and transport processes, genotype-fungus matching, and multi-environment field assessment. This evidence-aware framework can support microbiome-informed breeding, targeted inoculant development, and integrated management of wheat in salt-affected agroecosystems.

## Graphical abstract

**Figure:**
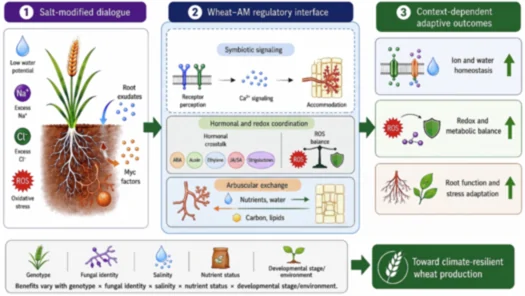

## 1. Introduction

Salinity is a major abiotic stress limiting wheat (*Triticum aestivum* L.) productivity worldwide, affecting over 20% of irrigated lands and threatening global food security.^1^ It imposes osmotic constraints, ionic imbalances from excessive Na⁺ and Cl^−^, and oxidative damage caused by reactive oxygen species (ROS). These stresses disrupt cellular homeostasis, impair photosynthesis, reduce nutrient uptake, and trigger maladaptive defenses.^2-4^ Wheat shows wide variation in salt tolerance, governed by complex interactions among osmotic adjustment, ion transport, redox balance, hormone signaling, and transcriptional regulation.^5-7^ Substantial progress has been made in identifying salt-tolerant germplasm, improving wheat cultivars, characterizing adaptive metabolites and genes, and applying molecular, genomic, breeding, and agronomic approaches.^8^ Nevertheless, the expression of tolerance remains sensitive to salinity intensity, developmental stage, soil heterogeneity, and genotype × environment interactions.^6^ ^,^ ^7^ ^,^ ^9^ Arbuscular mycorrhizal symbiosis should therefore be considered a complementary biological strategy rather than a replacement for established breeding and crop-management approaches.

Arbuscular mycorrhizal (AM) fungi form ancient mutualistic associations with most terrestrial plants, including cereals.^10^ ^,^ ^11^ AM symbiosis enhances water and nutrient uptake particularly phosphorus (P), while modulating stress perception, hormonal networks, and redox homeostasis.^12^ ^,^ ^13^ Direct studies in salt-stressed wheat and durum wheat show that AM colonization can improve K⁺/Na⁺ balance, P and micronutrient acquisition, plant water status, membrane stability, antioxidant regulation, biomass, and stress-responsive gene expression.^14^ ^,^ ^15^ These findings establish a consistent physiological and emerging transcriptomic basis for AM-mediated salinity tolerance in wheat. By contrast, the detailed molecular processes governing fungal recognition, symbiotic Ca^2+^ signaling, transcriptional accommodation, arbuscule development, and plant-to-fungus lipid transfer have been characterized principally in AM model plants and cereals other than wheat.^12^ ^,^ ^16^ These conserved mechanisms provide a robust framework for interpreting interactions between wheat and AM fungi but should not be considered fully validated in wheat under salinity.

Collectively, the evidence suggests that AM-associated benefits arise from coordination among AM fungal spore germination and growth, arbuscule-mediated exchange, ion and water transport, redox regulation, hormonal crosstalk, and host carbon (C) allocation.^12^ ^,^ ^17^ However, direct wheat studies mainly document physiological and transcriptional outcomes, whereas the molecular links connecting these responses are inferred largely from conserved mechanisms in other AM-compatible systems.

This review focuses on the symbiosis between wheat and AM fungi as one component of the wider wheat-associated microbiota. The outcome of this focal interaction emerges from reciprocal signaling and resource exchange and is not necessarily beneficial. Rather, the outcome depends on whether fungal contributions to nutrient acquisition, water relations, and stress protection compensate for the C and lipid resources supplied by the host. Wheat genotype, AM fungal identity, salinity intensity, nutrient availability, developmental stage, and environmental conditions can therefore shift the interaction from strongly beneficial to weak, neutral, or metabolically costly.^13^ ^,^ ^18^ ^,^ ^19^ Colonization percentage alone provides limited information about symbiotic function; depending on the study objective, it should be interpreted alongside one or more relevant indicators, such as arbuscule activity, nutrient transfer, extraradical hyphal development, host C expenditure, or plant performance.

Among the 11 primary studies directly examining AM-colonized wheat under salinity, nine primarily evaluated physiological, biochemical, nutritional, photosynthetic, agronomic, or yield-related responses, whereas only two included molecular analyzes. This limited molecular evidence reinforces the need to distinguish wheat-supported outcomes from mechanisms inferred from AM model plants and cereals other than wheat.

Here, we synthesize current knowledge of responses of AM-colonized wheat to salinity, from presymbiotic communication and fungal accommodation to ion homeostasis, redox regulation, metabolic adjustment, hormonal crosstalk, and stress adaptation. We explicitly differentiate direct evidence from AM-colonized wheat under salinity, wheat-specific evidence obtained outside the combined stress-symbiosis context, and mechanisms characterized in other cereals or AM model systems. Rather than presenting a fully resolved wheat-specific signaling network, we use these complementary evidence levels to distinguish conserved regulatory principles, wheat-supported adaptive outcomes, and priorities for causal validation. This evidence-aware framework can guide genotype-fungus matching, microbiome-informed breeding, inoculant development, and field-oriented management of wheat in salt-affected environments.

## 2. Literature search and evidence classification

This review was conducted as a structured narrative synthesis rather than a systematic review or meta-analysis. Literature searches were performed in the Web of Science Core Collection, Scopus, PubMed, and Google Scholar. No publication-date restriction was applied and literature search was completed on 14 July 2026. The principal search string combined “wheat” or “*Triticum aestivum*” or “*Triticum durum*” and “arbuscular mycorrhiza” or “arbuscular mycorrhizal fungi” or “AM fungi” and “salinity” or “salt stress” or “saline soil”. Additional searches incorporated mechanistic terms, including “root exudates,” “strigolactones,” “Myc factors,” “LysM receptor,” “common symbiosis signaling pathway,” “CCaMK,” “CYCLOPS,” “RAM1,” “arbuscule,” “ion transporter,” “HKT,” “SOS1,” “NHX,” “aquaporin,” “calcium,” “ROS,” “hormone,” “transcriptome,” “gene expression,” “C allocation,” and “lipid transfer.” Reference lists of relevant primary studies and reviews were also examined to identify additional publications.

Peer-reviewed primary studies were included when they investigated interaction between wheat and AM fungi under salinity, interaction between wheat and AM fungi under non-saline conditions, wheat salinity responses without AM colonization, or conserved AM mechanisms relevant to interpreting wheat responses. Review articles, including recent wheat-salinity syntheses, were used for conceptual background and identification of primary studies but were not counted as direct experimental evidence. Duplicate records, conference abstracts, theses, non-peer-reviewed material, studies involving non-AM fungal associations, and publications without clear relevance to wheat, salinity, or transferable AM mechanisms were excluded. Records were screened first by title and abstract and subsequently through full-text assessment.

Evidence was classified into four categories: direct evidence from AM-colonized wheat under salinity; wheat-AM evidence under non-saline conditions; wheat salinity evidence generated without AM colonization; and mechanistic evidence from other cereals or AM model plants. Within the direct wheat-AM-salinity category, physiological, biochemical, nutritional, and agronomic evidence was distinguished from molecular or transcriptomic evidence. Evidence was considered functionally causal only when a defined signaling component was evaluated through genetic perturbation, complementation, reporter analysis, protein localization or activity, dynamic ion or hormone measurements, or an equivalent experimental approach. The evidence status of the principal processes, together with the major wheat-specific knowledge gaps, is summarized in Table 1.

**Table 1.** Evidence classification of major processes in wheat-arbuscular mycorrhizal responses to salinity. The table distinguishes direct evidence from wheat under salinity, wheat evidence obtained outside the combined salinity-symbiosis context, mechanisms characterized in other cereals or AM model plants, and the principal wheat-specific knowledge gaps.

Process or mechanism	Wheat under salinity	Wheat under non-saline conditions	Other cereals or AM model plants	Main knowledge gap	References
Root exudates and strigolactones	Limited: colonization documented; exudation rarely measured	Limited wheat-specific evidence	Strong: fungal activation and hyphal branching established	Link wheat exudate profiles with AM fungal spore germination and growth under salinity	[14,20,21]
Myc-factor perception and common symbiosis pathway	Very limited: no direct functional validation	Candidate homologs identified	Strong: receptors, Ca^2+^ oscillations, CCaMK and CYCLOPS established	Validate pathway components and Ca^2+^ signatures in salt-stressed wheat	[16,22,23]
Arbuscule development and nutrient exchange	Moderate: colonization and nutrient benefits reported	C-for-nutrient exchange demonstrated	Strong: RAM1, RAD1, STR/STR2 and lipid-transfer pathways established	Measure arbuscule viability, nutrient flux and C cost	[15,19,24]
Na⁺/K⁺ homeostasis and ion transport	Strong physiological; limited causal evidence	Wheat HKT, SOS1 and NHX functions established	AM-associated transporter responses reported in crops	Resolve direct AM symbiosis effects on transporter localization and activity	[15,25,26]
Aquaporins and water relations	Moderate: improved water status; limited protein-level evidence	Limited AM-specific wheat evidence	Aquaporin regulation characterized in other plants	Identify PIP/TIP activity and hydraulic contribution in wheat	[27–29]
ROS and antioxidant regulation	Strong biochemical; moderate transcriptomic evidence	Redox benefits reported in wheat-AM systems	Strong: localized ROS functions established in AM models	Separate signaling ROS from oxidative damage	[15,17,30]
Hormonal crosstalk and defense balance	Limited to moderate: mainly indirect or transcriptomic evidence	Wheat salinity-hormone pathways established	Strong: AM regulation of ABA, auxin, ethylene, JA and SA established	Quantify hormone dynamics, sensitivity and response thresholds	[17,25,31]
C and lipid allocation	Limited: photosynthetic responses reported; fluxes rarely measured	Cultivar-dependent C exchange demonstrated	Strong: RAM2, STR/STR2, FatM and WRI5 functions established	Determine when nutrient return exceeds fungal C cost	[19,32,33]
Transcriptional and post-transcriptional regulation	Moderate transcriptomic; limited functional evidence	Wheat miRNAs and homoeologue responses characterized separately	AM-responsive regulators established in model systems	Validate cell- and homoeologue-specific regulatory functions	[17,34,35]
Genotype × fungus × environment effects	Moderate: context-dependent benefits documented	Cultivar variation demonstrated	Broad support across crop systems	Identify stable genotype-fungus combinations for field use	[19,36,37]

Note: Strong, replicated or functionally validated evidence; moderate, direct but mainly physiological, biochemical, or associative evidence; limited, sparse evidence without direct functional validation.

The final evidence set included 11 primary studies directly examining AM-colonized wheat under salinity. Of these, nine predominantly assessed physiological, biochemical, nutritional, photosynthetic, agronomic, or yield-related outcomes, whereas two included molecular-level analyzes: targeted gene-expression measurements and transcriptome-wide analysis (Table S1). Accordingly, mechanisms involving fungal-signal receptors, nuclear Ca^2+^ decoding, CCaMK/CYCLOPS signaling, GRAS-family regulators, arbuscule developmental control, and plant-to-fungus lipid transfer are treated as model-derived mechanistic frameworks and testable hypotheses rather than as pathways functionally validated in wheat under salinity (Table 1).

## 3. Establishment of wheat-AM symbiosis under salinity

**Figure 1.**
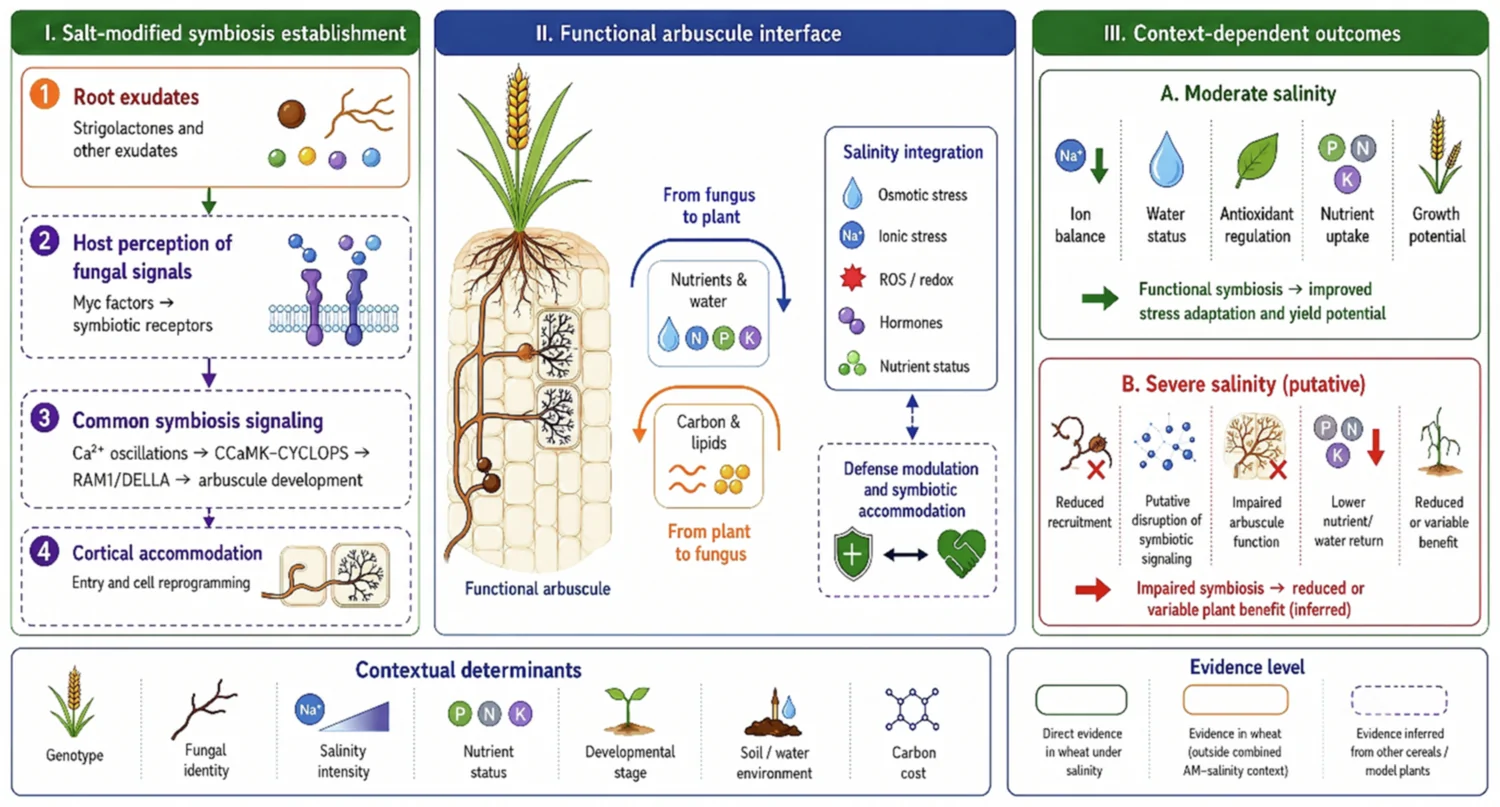
Framework for wheat-AM symbiosis establishment under salinity. Root exudates stimulate presymbiotic activity in AM fungi and fungal signals activate receptor-mediated common symbiosis signaling and downstream transcriptional regulators. Salinity-modulated hormonal and redox signals regulate root receptivity, defense tone, and arbuscule development. Functional arbuscules mediate nutrient and water delivery in exchange for host C and lipids. Moderate salinity supports symbiotic function and stress adaptation, whereas severe salinity impairs recruitment and arbuscule functionality.

Presymbiotic communication between wheat roots and AM fungi begins before physical contact and is mediated by reciprocal molecular signaling. AM symbiosis is subsequently established through recognition and contact between the partners, root penetration and colonization, cortical accommodation, and functional arbuscule development within root cortical cells (Figure 1).^38^ ^,^ ^39^ The principal components of this sequence have been functionally characterized in AM model plants and provide a strong mechanistic framework for wheat, although their regulation under combined wheat-AM-salinity conditions is less completely resolved. Salinity places additional constraints on this dialog because both partners must remain metabolically and signaling competent under low water potential, Na^+^ and Cl^−^ toxicity, nutrient limitation, and oxidative pressure.^13^ ^,^ ^40^ ^,^ ^41^ Establishment of AM symbiosis in wheat under salinity is therefore a regulated sequence involving presymbiotic communication, recognition and contact, root penetration and colonization, cortical accommodation, arbuscule development, and functional exchange rather than passive colonization.

### 3.1. Presymbiotic chemical communication under salinity

Presymbiotic communication between wheat roots and AM fungi occurs before physical contact and involves compounds released into the rhizosphere. Strigolactones are the best-characterized host-derived signals and stimulate AM fungal metabolic activity, spore germination in responsive fungal taxa, hyphal branching, and hyphal growth near receptive roots.^20^ ^,^ ^21^ Other root-exudate classes, including flavonoids and phenolics, sugars, amino acids, and organic acids, can modify the chemical and nutritional conditions of the rhizosphere, but their direct effects on AM fungi are not equally established and their signaling roles remain largely untested in salt-stressed wheat. Strigolactone biosynthesis involves the carotenoid isomerase D27, the carotenoid cleavage dioxygenases CCD7/MAX3 and CCD8/MAX4, and MAX1-type cytochrome P450 monooxygenases. Within the plant, D14/DAD2 receptor, and the MAX2/D3 F-box protein, together with associated repressors mediate strigolactone perception and downstream developmental responses.^42^ ^,^ ^43^ Exuded strigolactones, by contrast, act directly on AM fungi by stimulating presymbiotic metabolic activation and hyphal development. These functions are well known in model plants and cereals other than wheat and need to be directly validated in wheat under salt stress.

Available evidence provides only a qualified answer regarding the effect of salinity on presymbiotic chemical communication. Studies in non-wheat hosts indicate that salinity can modify strigolactone production, but they do not demonstrate that salinity alters the pre-contact chemical communication between wheat roots and AM fungi. P limitation generally enhances strigolactone biosynthesis and exudation.^43^ ^,^ ^44^ Whereas salinity may influence this response indirectly through changes in root metabolism, C availability, and AM fungal activity.^13^ ^,^ ^40^ ^,^ ^45^ However, direct wheat studies have not quantified strigolactone exudation under salinity and subsequently tested the resulting exudates for effects on AM fungal metabolic activation, spore germination, hyphal growth, or branching. Salinity-dependent modification of presymbiotic chemical communication in wheat should therefore be regarded as plausible but currently unvalidated.

Salinity may also alter the composition and abundance of flavonoids, phenolics, sugars, amino acids, organic acids, and other compounds released by wheat roots. Such changes could influence nutrient availability, rhizosphere redox conditions, and AM fungal activity. Nevertheless, direct effects of most of these compounds on AM fungal metabolism, spore germination, or hyphal development have not been demonstrated in salt-stressed wheat. They should therefore be described as potential modifiers of the rhizosphere environment rather than collectively treated as established presymbiotic signals.

### 3.2. Fungal signal perception and activation of the common symbiosis pathway

After perceiving host-derived signals, AM fungi increase hyphal branching and release symbiotic molecules.^20^ ^,^ ^21^ These include lipo-chitooligosaccharides and short-chain chitooligosaccharides, collectively referred to as Myc factors.^22^ ^,^ ^46^ ^,^ ^47^ Root surface receptor-like kinases perceive these signals, particularly LysM-domain receptor-like kinases, together with CERK1-like and SYMRK/DMI2-type components.^48^ ^,^ ^49^ Receptor classes such as LYK3/NFR1-like, NFP/NFR5-like, CERK1-like, and SYMRK/DMI2 are candidate components of early AM recognition, although their wheat-specific functions under salinity need further validation.

Recognition of AM fungal signals activates the common symbiosis signaling pathway, a conserved module required for intracellular fungal accommodation. This pathway includes nuclear-envelope and nucleoporin-associated components such as CASTOR, POLLUX/DMI1, NUP85, NUP133, and NENA. These components contribute to nuclear and perinuclear Ca^2+^ oscillations, which are decoded by CCaMK/DMI3 to activate CYCLOPS/IPD3 and downstream transcriptional programs required for fungal accommodation and arbuscule development.^16^ ^,^ ^50^ ^,^ ^51^ This pathway is well established in AM model plants and is likely conserved in cereals, but salinity-dependent Ca^2+^ signatures and pathway activation have not been mapped directly in wheat.

Under salinity, fungal recognition occurs against a background of elevated stress and defense signaling, which can restrict penetration and arbuscule formation when excessive.^31^ ^,^ ^40^ ^,^ ^41^ Selective immune adjustment is thus required for successful establishment of plants, instead of generalized defense suppression. Transcriptional regulation connects signal perception with symbiotic accommodation. NSP1, NSP2, RAM1, RAD1, and DELLA link strigolactone signaling, fungal accommodation, and arbuscule development.^16^ ^,^ ^52^ Other stress-responsive families including WRKY, NAC, MYB, AP2/ERF, bZIP, and bHLH are also involved in salinity and AM responses in wheat, but their direct roles in fungal accommodation have yet to be determined.

### 3.3. Salinity as a modifier of early symbiotic signaling

Salinity affects establishment of AM symbiosis at several physiological and molecular levels. High salt decreases soil water potential, thereby slowing root elongation and fungal hyphal growth.^40^ ^,^ ^41^ It also alters Ca^2+^, ROS and hormonal signals which overlap with fungal recognition and accommodation pathways.^31^ ^,^ ^53^ Under mild to moderate salinity, AM-colonized wheat commonly maintains greater P acquisition, water status, Na⁺ exclusion, K⁺ retention, antioxidant capacity, and growth than non-colonized plants.^40^ ^,^ ^45^ These outcomes indicate that recruitment and functional exchange can persist while stress remains physiologically manageable. Severe salinity has the opposite effect, suppressing colonization and symbiotic performance by reducing C supply to the fungus, impairing root metabolism, intensifying defense responses, and limiting fungal accommodation.^32^ ^,^ ^53^ The transition from beneficial to weak or costly symbiosis is therefore evident physiologically, although its regulatory checkpoints remain unresolved (Figure 1).

Salinity-dependent changes in strigolactones, ABA, auxin, ethylene, jasmonate, salicylate, and ROS influence root receptivity, defense tone, and fungal entry. Controlled auxin, ROS, and defense signaling can support cortical accommodation, whereas sustained ethylene, salicylate-associated defense, or oxidative stress may shift the interaction toward incompatibility. These relationships are mechanistically supported mainly by model systems; their spatial and temporal coordination in wheat remains incompletely resolved.^31,44,54–58^

Transcriptomic studies in mycorrhizal wheat and durum wheat under salinity report changes in genes related to signal transduction, ion transport, antioxidant metabolism, cell-wall remodeling, vesicle trafficking, and TF families such as WRKY, NAC, MYB, and AP2/ERF.^14^ ^,^ ^17^ These patterns support coordinated stress-symbiosis reprogramming rather than independent activation of salt and AM pathways, although differential expression alone does not establish causality.

### 3.4. Formation of functional arbuscules and bidirectional signaling interfaces

After successful recognition and root entry, AM fungi develop intracellular arbuscules within cortical cells.^39^ These structures are surrounded by the plant-derived peri-arbuscular membrane, which acts as the critical interface for the bidirectional exchange of nutrients, C, lipids, and signaling molecules.^59^ ^,^ ^60^ Arbuscule formation is a highly organized process that necessitates extensive host cell reprogramming, membrane trafficking, coordinated lipid biosynthesis, phosphate transport, and symbiosis-specific transcriptional activation.^24^ ^,^ ^50^

GRAS transcriptional regulators and lipid-biosynthesis and transfer components are central to arbuscule branching, maintenance, and developmental turnover.^16^ ^,^ ^33^ Transporters such as STR and STR2 are localized to the peri-arbuscular membrane and are required for proper arbuscule development, supporting the importance of plant-to-fungus lipid-related exchange during functional symbiosis.^24^ ^,^ ^61^ These mechanisms are established mainly in AM model plants and provide candidate pathways for functional testing in wheat. In salt-stressed wheat, improved nutrient acquisition, ion balance, water relations, antioxidant status, and transcriptional responses indicate that active arbuscules contribute to stress adaptation.^15^ ^,^ ^17^ However, direct regulation of aquaporins, proton pumps, and ion transporters at the wheat peri-arbuscular interface remains less resolved than these physiological outcomes.

Colonization percentage alone does not establish functional symbiosis. The presence of fungal structures should be distinguished from arbuscule abundance, branching, viability, nutrient-transfer activity, extraradical hyphal development, and host C investment. Highly colonized roots may provide little protection when arbuscules are senescent, nutrient transfer is weak, or fungal C demand exceeds the returned benefit. Severe salinity can accelerate arbuscule decline by reducing photosynthesis and C availability or by intensifying oxidative and defense signaling.^32^ ^,^ ^53^ Arbuscule functionality, rather than colonization alone, is therefore a central determinant of AM effectiveness in salt-stressed wheat. Overall, establishment under salinity proceeds through linked checkpoints involving root exudation, fungal-signal perception, common symbiosis signaling, cortical accommodation, and functional arbuscule development. Direct wheat evidence supports the physiological and transcriptional outcomes of this sequence, whereas model systems provide the molecular framework for fungal recognition, Ca^2+^ decoding, transcriptional accommodation, and lipid transfer.

## 4. AM modulation of wheat stress perception under salinity

**Figure 2.**
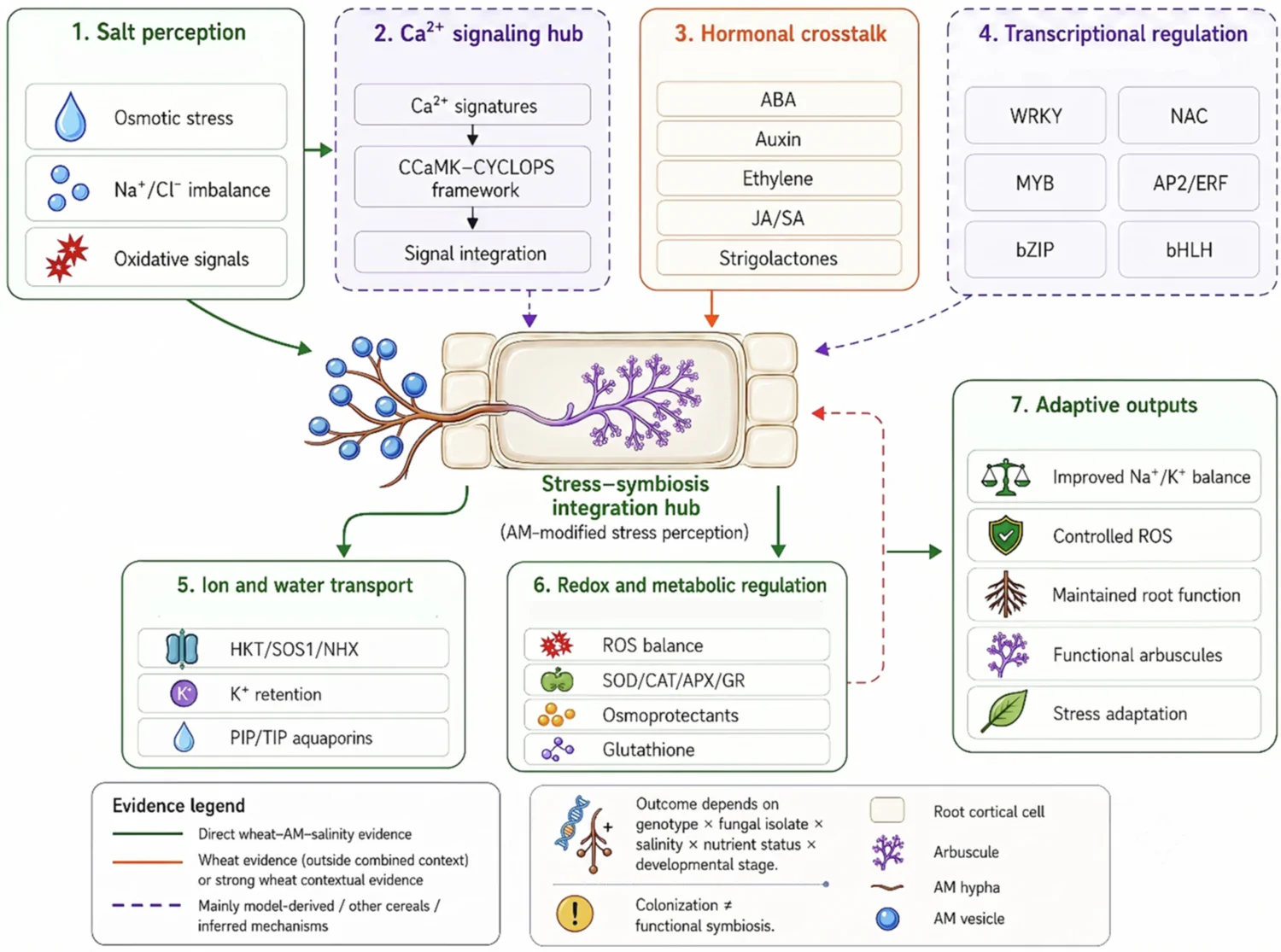
Evidence-aware model of stress perception modified by AM symbiosis in wheat under salinity. Salt perception converges with Ca^2+^, hormonal, and transcriptional signaling to coordinate ion and water transport, redox and metabolic regulation, and adaptive physiological outputs. Functional arbuscules within cortical cells integrate nutrient exchange and stress adaptation. The arrangement highlights the coordinated network by which AM fungi modulate stress perception, signaling pathways, and root adaptation under moderate and severe salinity.

Salinity stress is perceived by wheat through overlapping osmotic, ionic, oxidative, calcium-dependent, and hormonal signals that alter root physiology, membrane transport, transcriptional activity, and defense status. Studies in AM-colonized wheat show that improved plant water status, membrane stability, ion balance, antioxidant regulation, and stress-responsive gene expression.^14^ ^,^ ^15^ These responses reflect coordinated adjustment of osmotic and ionic regulation, Ca^2+^-ROS signaling, hormonal networks, transcriptional control, and fungal accommodation rather than nutrient acquisition alone (Figure 2).^17^ ^,^ ^62^ Direct wheat studies strongly support the physiological and transcriptomic outcomes of this adjustment, whereas the detailed signaling connections are inferred partly from conserved mechanisms established in other cereals and AM model plants. Together, the available evidence indicates that AM fungi act as biological modulators of stress-signaling thresholds, helping wheat maintain adaptive signaling and functional symbiosis without excessive activation of damage responses.

### 4.1. Salt perception: osmotic, ionic, and oxidative signals

Reduced soil water potential initially restricts water uptake, turgor, and growth, whereas prolonged exposure causes Na⁺ and Cl^−^ accumulation, K⁺ loss, membrane disruption, and secondary oxidative stress.^63^ ^,^ ^64^ Although high ROS levels damage proteins, lipids, nucleic acids, and membranes, controlled ROS production also functions as a signal for antioxidant activation, cell-wall remodeling, and stress-responsive gene expression.

AM symbiosis modifies these primary stress signals before they develop into irreversible injury. Mycorrhizal wheat commonly maintains greater root water status, membrane stability, P and micronutrient acquisition, lower Na⁺ uptake, greater K⁺ retention, and a higher K^+^/Na^+^ ratio under salinity than non-colonized wheat.^14^ ^,^ ^15^ These effects reduce the osmotic and ionic burden perceived by the host. Improved membrane stability in mycorrhizal durum wheat also limits ion leakage and ROS amplification, thereby connecting physiological protection with reduced downstream stress signaling.^14^ ^,^ ^15^

At the molecular level, this buffering effect is linked with changes in ion- and water-transport pathways involving HKT, SOS1, NHX, AKT/KUP/HAK-type K⁺ transporters, PIP/TIP aquaporins, and proton pumps such as H⁺-ATPases and H⁺-pyrophosphatases (Table 2).^25^ ^,^ ^65^ These systems have established functions in wheat salinity tolerance, while transcriptomes of AM-colonized wheat indicate broader modification of transport, redox, and regulatory pathways involving WRKY, NAC, MYB, and AP2/ERF factors.^14^ ^,^ ^17^ However, direct AM-dependent regulation of individual transporter proteins or transcription factors in salt-stressed wheat remains less resolved than the resulting physiological improvements. Thus, AM fungi appear to lower the effective stress burden and adjust the threshold for costly damage responses, rather than simply repairing injury after it occurs.

**Table 2.** Evidence status of major signaling and regulatory modules proposed in wheat-arbuscular mycorrhizal responses to salinity. Direct evidence refers to wheat-AM studies under salinity; partial evidence includes physiological, biochemical, or transcriptomic associations; model-derived mechanisms are established mainly in other cereals or AM model plants and are presented as mechanistic frameworks rather than validated wheat pathways.

Module	Key components	Evidence status in wheat-AM-salinity	Main interpretation	References
Pre-symbiotic signaling	Strigolactones, phenolics, sugars, organic acids	Model-derived; limited wheat evidence	Proposed control of AM fungal spore germination and growth	[12,20,44,50,66]
Fungal signal perception	Myc factors, LysM-RLKs, CERK1-like, SYMRK/DMI2	Model-derived	Conserved recognition framework	[22,67–69]
Common symbiosis signaling	Ca^2+^ oscillations, CCaMK/DMI3, CYCLOPS/IPD3	Validated in AM models; unvalidated in salt-stressed wheat	Links perception with cortical reprogramming	[16,70–73]
Symbiotic transcriptional control	NSP1/2, RAM1, RAD1, DELLA, WRI5	Mainly model-derived	Controls accommodation and arbuscule development	[16,52,74–76]
Salt-induced Ca^2+^ signaling	CBL-CIPK, CDPK/CPK, CaM/CML, SOS pathway	Established in wheat salinity biology; AM-specific role unresolved	Couples salt perception with transport regulation	[25,77]
ROS/redox regulation	RBOH, SOD, CAT, APX, GR	Direct biochemical; limited causal evidence	Maintains ROS balance and antioxidant protection	[15,78]
Hormonal crosstalk	ABA, auxin, ethylene, JA, SA, strigolactones	Partial wheat evidence; network mainly inferred	Coordinates root plasticity, defense, and compatibility	[17,44,56,79]
Ion homeostasis	HKT, SOS1, NHX, AKT1, HAK/KUP/KT	Strong physiological; limited transporter-level validation	Improves Na⁺/K⁺ balance	[15,80]
Water and osmotic regulation	PIP/TIP, P5CS, BADH, SWEET, SUT/SUC	Moderate physiological; limited functional evidence	Supports water status and osmotic adjustment	[14,78]
Arbuscule nutrient exchange	PT4/PT11-like, PHT1, AMT, NRT	Nutritional benefits shown; direct flux evidence limited	Links nutrient delivery with stress adaptation	[15,81–83]
C and lipid transfer	RAM2, STR/STR2, FatM, DIS, SWEET	Mainly model-derived	Determines symbiotic C cost-benefit	[33,84–87]
Stress-responsive TFs	WRKY, NAC, MYB, bZIP, AP2/ERF, bHLH	Transcriptomic; limited causal evidence	Supports a distinct stress-symbiosis state	[14,17]
Post-transcriptional regulation	miRNAs, alternative splicing	Very limited combined-context evidence	Proposed tissue- and homoeologue-specific control	[34,88–90]
Metabolic regulation	Proline, sugars, glycine betaine, glutathione, phenolics	Direct biochemical; limited pathway validation	Supports osmotic and redox adjustment	[17,78]

### 4.2. Calcium, ROS, and hormone networks as integrated signaling hubs

Ca^2+^, ROS, and hormones form interconnected signaling hubs linking salt perception with fungal accommodation (Figure 2). During establishment of AM symbiosis, fungal signals activate the common symbiosis signaling pathway, generating nuclear and perinuclear Ca^2+^ oscillations that are decoded by CCaMK/DMI3 and CYCLOPS/IPD3 to induce transcriptional programs for fungal accommodation and arbuscule development (Table 2).^16^ ^,^ ^51^ This pathway is well characterized in AM model plants and provides the principal mechanistic framework for interpreting symbiotic Ca^2+^ signaling in wheat. Ca^2+^ also controls membrane stabilization, ion transporter activity, Na^+^/K^+^ regulation and stress responsive gene expression under salinity.^65^ ^,^ ^91^ How salt- and symbiosis-induced Ca^2+^ signatures differ in their frequency, duration, subcellular location, and downstream decoding in salt-stressed wheat remains unresolved.

ROS provide a second level of integration. Wheat studies show that AM colonization improves antioxidant capacity and oxidative-stress status, whereas evidence from model systems indicates that localized ROS supports fungal entry, cell-wall remodeling, and arbuscule function.^15^ ^,^ ^55^ Enzymatic and non-enzymatic systems, represented by SOD, CAT, APX, glutathione metabolism, and redox-sensitive proteins, help maintain this balance. Hence, AM symbiosis does not just inhibit ROS production, but rather allows a redox window where ROS can be used for signaling, while preventing its amplification of oxidative injury.

This Ca^2+^-ROS system is modulated by hormonal networks. ABA influences osmotic regulation and water transport, auxin shapes root and cortical development, ethylene, jasmonate, and salicylate regulate defense tone, and strigolactones link P status with AM fungal spore germination, hyphal branching and growth.^31^ ^,^ ^92^ AM colonization can alter hormone levels, sensitivity, and crosstalk, which can alter the magnitude and duration of downstream stress responses.^79^ ^,^ ^92^ Because these effects vary with wheat genotype, fungal identity, salinity intensity, and developmental stage, hormonal integration helps explain the context dependence of AM-associated benefits.

### 4.3. Balancing defense activation and symbiotic compatibility

AM fungi are beneficial partners, but their intracellular colonization requires wheat to maintain immune control without rejecting the fungus. Salinity complicates this balance because salt stress triggers defense-like responses such as ROS bursts, ethylene accumulation, SA signaling, cell-wall reinforcement, pathogenesis-related genes, and defense-associated transcription factors.^63^ ^,^ ^64^ Although these responses protect against stress injury, excessive activation can restrict fungal penetration, arbuscule formation, and nutrient exchange. Therefore, AM-mediated salt tolerance is not based on general defense suppression, but on selective defense modulation.^93^

AM symbiosis contributes to this balance by regulating ROS production, hormone crosstalk, and defense-related gene expression. In AM model systems, localized ROS production facilitates controlled cell-wall modification and redox signaling at fungal entry sites, while moderate JA signaling can maintain defense readiness without blocking fungal accommodation.^31^ ^,^ ^54^ By contrast, excessive ROS accumulation, sustained ethylene signaling, or strong SA-associated defense can reduce fungal compatibility. ABA further links osmotic protection with growth-defense regulation.^53^ ^,^ ^92^ Together, these conserved interactions provide a plausible basis for the protective yet permissive state observed in mycorrhizal wheat.

At the molecular level, this balance is coordinated by stress-related transcription factors and symbiosis regulators. WRKY, NAC, MYB, AP2/ERF, bZIP and bHLH families participate in defense, redox, cell-wall, and hormonal responses, while transcriptomic studies show differential regulation of WRKY, NAC, and MYB genes in mycorrhizal durum wheat under salinity.^14^ ^,^ ^17^ These findings support coordinated transcriptional adjustment, although differential expression alone does not establish the direct function of individual transcription factors in fungal accommodation. This balance varies with wheat genotype, fungal identity, salinity level, P availability, and colonization stage. Under moderate stress, wheat can maintain C supply, functional arbuscules, and controlled defense signaling.^32^ ^,^ ^53^ Under severe salinity, damage signaling intensifies, host C availability declines, and the symbiotic benefit weakens.

### 4.4. Transcriptional and post-transcriptional regulation of AM-mediated salt responses

The downstream outcome of stress perception modified by AM symbiosis is coordinated transcriptional and post-transcriptional reprogramming.^17^ ^,^ ^94^ Wheat roots must activate protective responses while maintaining the fungal-accommodation, nutrient-exchange, and C-allocation programs required for functional symbiosis.^16^ ^,^ ^52^ In AM model plants, CCaMK/DMI3, CYCLOPS/IPD3, NSP1, NSP2, RAM1, RAD1, DELLA, and WRI5 connect fungal recognition and accommodation with arbuscule development, C allocation, and nutrient-exchange programs.^16^ ^,^ ^95^ These conserved regulators provide a mechanistic framework for wheat, although their coordinated activity under combined AM colonization and salinity remains unresolved. At the same time, salt-responsive genes such as *HKT, SOS1, NHX, PIP/TIP* aquaporins, *P5CS, BADH,* antioxidant genes, and ABA/ethylene-responsive genes determine whether roots maintain ionic and osmotic stability.^25^ ^,^ ^65^ Thus, AM-colonized wheat under salinity enters a distinct regulatory state in which stress-protective and symbiotic-accommodation genes are coordinately expressed, rather than simply forming a stronger salt-response program (Table 2).

Transcriptomic evidence in mycorrhizal durum wheat supports this interpretation, showing that AM colonization can alter salt-responsive gene expression.^17^ These expression patterns are consistent with a lower effective stress burden in colonized plants and less activation of damage pathways that restrict growth, C allocation, and fungal accommodation.^14^ ^,^ ^17^ Nevertheless, transcriptomic associations should be complemented by protein localization, enzyme activity, mutant or gene-editing analyzes, and measurements of arbuscule function to establish causality.

Post-transcriptional mechanisms further refine this regulatory state. Small RNAs, alternative splicing, RNA stability, RNA methylation, and translational control can adjust gene expression rapidly and tissue-specifically.^96^ ^,^ ^97^ This is especially relevant in polyploid wheat, where homoeologous gene copies may contribute differently to stress and symbiotic responses. MicroRNAs such as miR160, miR164, miR169, and miR398 participate in root development, hormonal regulation, and antioxidant responses.^96^ ^,^ ^98^ They therefore represent plausible regulators of AM-modified salt responses in wheat. However, wheat-specific evidence for small RNA regulation during combined AM symbiosis and salt stress remains limited. Similarly, alternative splicing and homoeologue-specific regulation are established features of wheat stress responses, but their roles in fungal accommodation under salinity remain largely hypothetical. Overall, AM-mediated rewiring of wheat stress perception is a multi-layer regulatory process rather than a single protective effect. Direct wheat evidence demonstrates substantial physiological and transcriptomic modification under salinity, while conserved pathways from AM model systems provide testable mechanisms for how these responses may be coordinated.

## 5. Integrated ion homeostasis, nutrient signaling, and membrane transport in wheat-AM symbiosis under salinity

Salt tolerance in wheat depends on coordinated ion equilibrium, nutrient acquisition, water movement, membrane energization, and redox stability. Arbuscular mycorrhizal symbiosis supports these processes through changes at the soil-root interface, arbuscular nutrient exchange, ion transporter activity, aquaporin-mediated water flow, and stress-responsive metabolism.^60^ ^,^ ^62^ In saline soils, excessive Na⁺ and Cl^−^ disturb K⁺ retention, P availability, nitrogen (*N*) assimilation, micronutrient balance, membrane potential, and root hydraulic conductance. AM fungi mitigate these limitations by expanding the absorptive zone with extraradical hyphae, improving nutrient and water acquisition, and supporting membrane function. Direct studies in mycorrhizal wheat strongly support improvements in ion balance, nutrient status, membrane stability, and water relations, whereas detailed AM-dependent regulation of individual transport proteins is understood partly from wheat salinity biology and other AM-compatible crop systems.^15^ ^,^ ^25^ ^,^ ^53^ Thus, tolerance mediated by AM symbiosis is a result of coordination of rhizosphere acquisition, cellular transport, membrane energization and arbuscular exchange, not just an increase in mineral uptake.

### 5.1. AM regulation of Na⁺ exclusion, K⁺ retention, and cellular ion balance

Disruption of Na⁺/K⁺ homeostasis is one of the most damaging effects of salinity in wheat. Excess Na⁺ competes with K⁺ for uptake and binding sites, disturbs membrane potential, inhibits enzyme activity, and disrupts metabolism. Thus, wheat tolerance depends on limiting Na^+^ uptake, retrieval of Na^+^ from the xylem, maintenance of cytosolic K^+^, and compartmentation of Na^+^ into vacuoles or less sensitive tissues.^4^ ^,^ ^25^ ^,^ ^26^ AM symbiosis often improves this balance by reducing Na⁺ accumulation, supporting K⁺ retention, and increasing the K⁺/Na⁺ ratio in salt-stressed wheat.^14^ ^,^ ^15^ These effects likely reflect coordinated changes in rhizosphere ion activity, membrane selectivity, transporter regulation, nutrient-supported energy metabolism, and redox protection rather than growth dilution alone.

HKT1;4 and HKT1;5-type transporters in wheat are associated with Na⁺ retrieval from the xylem and restriction of shoot Na⁺ accumulation.^26^ ^,^ ^99^ SOS1 mediates plasma-membrane Na⁺/H⁺ exchange and contributes to Na⁺ efflux and tissue-level Na⁺ distribution, whereas tonoplast NHX antiporters sequester Na⁺ into vacuoles to reduce cytosolic toxicity.^25^ ^,^ ^91^ K⁺ uptake and retention involve AKT1 and HAK/KUP/KT systems, while H⁺-ATPases and H⁺-pyrophosphatases generate the proton motive force required for secondary ion transport.^100^ ^,^ ^101^ These functions are well established in wheat salinity tolerance, and altered transporter-associated expression in mycorrhizal wheat is consistent with their contribution to AM-mediated ion regulation. However, direct AM symbiosis effects on transporter localization, phosphorylation, and ion-flux activity remain poorly resolved.

AM-induced improvements in P nutrition may support ATP-dependent proton pumping and secondary Na⁺ transport.^25^ ^,^ ^102^ Improved N and sulfur (S) status further protect ion gradients by supporting osmolyte synthesis, amino acid metabolism, and glutathione-based redox buffering.^78^ ^,^ ^103^ The extraradical hyphal network adds an external layer of ion regulation by extending nutrient and water acquisition beyond the root depletion zone and altering rhizosphere structure, nutrient availability, and ion diffusion patterns.^53^ ^,^ ^59^ ^,^ ^60^ AM-mediated Na⁺/K⁺ homeostasis therefore combines external rhizosphere buffering with internal Na⁺ exclusion, vacuolar sequestration, K⁺ retention, and nutrient-supported membrane energization.

### 5.2. Nutrient signaling at the arbuscular interface: *P*, *N*, S, and micronutrients

The arbuscular interface is the central site of nutrient exchange between AM fungi and wheat cortical cells. The plant-derived peri-arbuscular membrane (PAM) is a specialized exchange surface where plant C and lipids are exchanged for fungal-delivered nutrients.^33^ ^,^ ^104^ P is the best-characterized nutrient. Salinity reduces P availability through changes in soil chemistry, root growth, and nutrient mobility. AM fungi partly compensate for these limitations by exploring soil beyond the root depletion zone, extending the absorptive surface area, and activating mycorrhiza-associated uptake pathways.^15^ ^,^ ^105^

Mycorrhiza-inducible transporters and specialized H^+^-ATPases are involved in transfer of phosphates at the arbuscular interface. In cereals, PT4/PT11-like transporters are essential; wheat contains functionally related PHT1 members.^105^ ^,^ ^106^ These conserved systems provide a mechanistic basis for improved P acquisition in mycorrhizal wheat, although transporter-specific regulation under combined AM colonization and salinity remains incompletely resolved. Because phosphate transfer depends on both active arbuscules and proton-gradient maintenance, symbiotic P delivery is also an indicator of functional exchange rather than colonization alone.^15^ ^,^ ^106^ ^,^ ^107^

N, S, and micronutrients also contribute to AM-mediated salt tolerance. N is used for amino acid biosynthesis, osmolyte production, and protein turnover, which likely involves coordinated regulation of AMT and NRT transporters in wheat. S is essential for production of cysteine, methionine and glutathione, which are related to S nutrition, antioxidant defense and redox buffering.^108^ ^,^ ^109^ Micronutrients such as Zn, Cu, Fe, and Mn serve as essential cofactors for antioxidant enzymes like superoxide dismutases and peroxidases.^110^ Thus, AM mediated improvement in these nutrients can enhance antioxidant capacity, membrane stability, and metabolic recovery in salt stressed wheat. However, the magnitude of these benefits is conditional, depending on particular combinations of wheat genotype, fungal species, and soil environment.

Nutrient limitation promotes ROS accumulation, weakens membrane integrity, and constrains ATP-dependent transport, whereas improved acquisition can stabilize ion gradients and redox balance.^15^ ^,^ ^62^ Thus, the arbuscule is not only the site of nutrient transfer, but acts as an interface between the fungus' nutrient acquisition and the host's energy status, ion regulation, C allocation, and stress adaptation.^59^ ^,^ ^60^ Nevertheless, arbuscule abundance should not be assumed to indicate equivalent nutrient-transfer activity; direct measurements of symbiotic fluxes remain necessary to establish functional exchange.

### 5.3. Transporter and aquaporin regulation: HKT, SOS1, NHX, H⁺-ATPases, and water channels

The activity of the ion-transport systems described above depends on membrane energization, protein localization, and tissue-specific regulation. H⁺-ATPases and H⁺-pyrophosphatases establish the electrochemical gradients that energize Na⁺ extrusion, vacuolar sequestration, nutrient uptake, and pH control.^25^ ^,^ ^91^ ^,^ ^111^ AM colonization affects the expression of genes associated with Na⁺ transport, K⁺ retention, vacuolar sequestration, nutrient uptake, and water-channel regulation.^14^ ^,^ ^17^ These responses are consistent with the improved ion balance observed in mycorrhizal wheat, but expression changes should not be equated automatically with transporter activity. Functional regulation also involves phosphorylation, vesicle trafficking, membrane localization, turnover, lipid environment, and proton-gradient maintenance.^25^ ^,^ ^65^ Active Na⁺ extrusion and vacuolar sequestration thus require not only transcripts but also functional proton pumps, membrane integrity, ATP availability, and redox balance.^91^ ^,^ ^102^

Aquaporins link osmotic adjustment with ion transport. The plasma membrane intrinsic proteins and tonoplast intrinsic proteins control water permeability, root hydraulic conductivity, vacuolar water flux, and osmotic balance.^27^ ^,^ ^112^ AM fungi support water uptake by expanding the absorptive network via extraradical hyphae and modulating aquaporin expression or activity. Aquaporin gating is regulated by interacting hormonal, Ca^2+^, redox, phosphorylation, pH, and membrane-trafficking signals.^113^ ^,^ ^114^ Under mild to moderate salinity, AM-associated aquaporin regulation can support root hydraulic conductance, tissue hydration, and turgor. Under severe stress, selective channel closure may become equally important by limiting uncontrolled water movement and preserving cellular integrity.^27^ ^,^ ^28^ ^,^ ^112^ Hence, AM symbiosis effects should not be interpreted as uniform enhancement of transport. Rather, the symbiosis adjusts water flow and membrane transport according to stress intensity, tissue demand, nutrient status, and the metabolic requirements of the fungal partner.

### 5.4. Membrane signaling integration: linking ion transport, osmotic adjustment, redox balance, and stress perception

Ion, nutrient, and water transport collectively determine the stress burden perceived by wheat. Na^+^ accumulation in the cytosol affects the metabolism, membrane potential and redox balance, while Na^+^ exclusion, vacuolar sequestration, K^+^ retention, and regulated water flow stabilize cellular function.^3^ ^,^ ^7^ ^,^ ^27^ ^,^ ^115^ AM symbiosis reinforces this coordination by coupling extraradical resource acquisition and arbuscular nutrient exchange with proton-pump activity, membrane integrity, and aquaporin regulation.^15^ ^,^ ^62^ ^,^ ^102^ ^,^ ^107^ P supports cellular energy metabolism, N and S sustain osmolyte and glutathione production, and micronutrients maintain redox-active enzymes. These indirect nutritional effects can be as important as direct regulation of individual transporters.

Several TF families coordinate this integrated response. NAC, WRKY, MYB, bZIP, AP2/ERF, and bHLH regulators influence salt tolerance, antioxidant defense, hormone signaling, transporter function, and root adaptation. Changes in these TFs under AM could regulate the expression of genes involved in *HKT, SOS1, NHX, PIP/TIP, PHT1*, antioxidant genes, and osmolyte-biosynthesis genes including *P5CS and BADH*.^17^ ^,^ ^25^ Transcriptomic studies support coordinated modification of transporter, nutrient, aquaporin, antioxidant, and regulatory pathways in mycorrhizal wheat, although direct transcription factor-target relationships remain to be established. These findings indicate that AM-associated tolerance depends on synchronized physiological regulation rather than isolated enhancement of Na⁺ transport, nutrient uptake, or water movement.

The improved K⁺/Na⁺ ratio, nutrient status, membrane stability, and water relations observed in mycorrhizal wheat provide strong evidence of an integrated physiological benefit.^14^ ^,^ ^15^ However, the relative contributions of direct transporter regulation, fungal nutrient delivery, altered root development, and growth-related dilution remain to be distinguished.

## 6. Integrated redox and metabolic signaling in wheat-AM symbiosis under salinity

Arbuscular mycorrhizal symbiosis modifies salinity-induced oxidative and metabolic disturbances through coordinated regulation of ROS signaling, antioxidant capacity, C allocation, osmoprotectant metabolism, nutrient-dependent redox buffering, and arbuscule maintenance.^15^ ^,^ ^24^ Together, these processes form an integrated redox-metabolic network that supports root growth, membrane integrity, fungal accommodation and stress-adaptive metabolism. Tolerance mediated by AM symbiosis reflects coordinated metabolic reprogramming rather than antioxidant activation or osmolyte accumulation alone.^15^ ^,^ ^17^ Direct studies in mycorrhizal wheat strongly support improvements in antioxidant status, osmolyte metabolism, membrane protection, and C-related performance, whereas the spatial regulation of ROS and the molecular control of C and lipid transfer are understood largely from AM model systems.

### 6.1. ROS signaling and antioxidant buffering

ROS act both as adaptive signals and as sources of oxidative injury in salt-stressed wheat. Under controlled conditions, ROS such as hydrogen peroxide and superoxide regulate Ca^2+^ channels, MAPK cascades, hormone signaling, ion transport, antioxidant genes, and cell-wall remodeling.^116^ ^,^ ^117^ Respiratory burst oxidase homologs (RBOHs) generate apoplastic ROS that participate in stress, developmental, and plant-microbe signaling. MAPK cascades, calcium-dependent protein kinases, and redox-sensitive transcription factors then convert ROS changes into gene-expression responses. When production exceeds antioxidant capacity, ROS cause lipid peroxidation, protein and nucleic-acid oxidation, photosynthetic impairment, and membrane leakage.^118^ ^,^ ^119^

AM symbiosis reshapes this response by helping wheat maintain a functional redox window. Localized ROS can contribute to cell wall loosening, controlled defense activation and fungal accommodation at fungal entry and during the development of the arbuscule.^54^ ^,^ ^55^ These localized functions are particularly well established in AM model systems. Excessive ROS, by contrast, can restrict fungal penetration, damage cortical cells, and accelerate arbuscule degeneration. In wheat, AM-associated redox regulation is supported by reduced lipid peroxidation, improved membrane stability, coordinated antioxidant activity, and altered stress-responsive transcription.^14^ ^,^ ^15^ AM-mediated redox control should therefore not be interpreted as uniform ROS suppression. Effective symbiosis limits damaging accumulation while retaining localized ROS required for signaling and fungal accommodation.^54^ ^,^ ^117^ Consequently, low bulk ROS content alone is insufficient to demonstrate functional redox regulation, particularly when spatial and cell-specific ROS dynamics remain unresolved in wheat.

AM-mediated salinity tolerance is associated with coordinated activation of enzymatic and non-enzymatic antioxidant systems. SOD is responsible for the conversion of superoxide to hydrogen peroxide, while CAT, APX, peroxidases, and the ascorbate-glutathione cycle control hydrogen peroxide and cellular redox pools.^15^ ^,^ ^120^ Thioredoxins, peroxiredoxins, ascorbate, glutathione, phenolics, flavonoids, carotenoids, and tocopherols provide additional redox buffering and membrane protection.^121^ ^,^ ^122^ AM fungi strengthen these antioxidant networks directly via symbiosis related signaling and indirectly via improved nutrition.^15^ ^,^ ^29^ N and S are involved in synthesis of cysteine, methionine, and glutathione. P supports ATP production and phosphorylation-dependent signaling. Micronutrients like Zn, Cu, Fe, Mn, and Ni act as cofactors for antioxidant and metabolic enzymes.^108^ ^,^ ^110^ Antioxidant performance is therefore closely linked to nutrient status and functional arbuscular exchange.

Antioxidant activity is not an adequate measure of tolerance. High enzyme activity may indicate high oxidative stress rather than good protection. In contrast, moderate but coordinated antioxidant activation indicate better stress buffering.^123^ ^,^ ^124^ Similarly, decreased antioxidant activity could be due to a decrease in the oxidative challenge or a loss of antioxidant defense, depending on other physiological responses. Interpretation should therefore integrate ROS abundance, lipid peroxidation, membrane leakage, glutathione and ascorbate redox status, ion balance, arbuscule activity, and plant performance to distinguish genuine stabilization from stress-induced antioxidant overactivation.

### 6.2. C allocation and metabolic cost-benefit balance of symbiosis

The outcome of AM symbiosis in wheat under salinity is context dependent and may be beneficial, neutral, or metabolically costly, depending on whether fungal contributions compensate for the host’s C investment. The host plant provides photosynthetically fixed C for fungal growth, maintenance of arbuscules, and the development of extraradical hyphae.^24^ ^,^ ^84^ In AM model systems, sucrose metabolism, hexose transport, SWEET-type sugar transporters mediate C delivery, while lipid-transfer genes, including *RAM2/GPAT, STR, STR2, FatM,* and *DIS* are essential for exporting fatty acids from the plant to the fungus.^12^ ^,^ ^33^ Symbiotic regulators, such as RAM1, WRI5, DELLA, and CYCLOPS/IPD3 coordinate the development of arbuscule with C allocation and lipid metabolic reprogramming.^16^ ^,^ ^125^ These molecular mechanisms are well established in AM model plants and provide a strong framework for investigating C and lipid exchange in wheat, although their regulation under salinity remains incompletely resolved.

Under salinity stress, wheat must partition C among competing metabolic demands: maintenance respiration, root repair, osmolyte biosynthesis, Na⁺ extrusion, vacuolar sequestration, antioxidant regeneration, and fungal support. AM colonization enhances P and N acquisition, water status, alleviates Na^+^ toxicity, and stabilizes redox balance, allowing wheat plant to maintain photosynthetic efficiency and sustain C flow to the fungus.^45^ ^,^ ^126^ The exchange is mutually beneficial when the returns in nutrients and water from the fungus are greater than the host's C costs.^32^

However, under severe or prolonged salinity, reduced photosynthesis affects C supply, which affects the functionality of arbuscules.^24^ ^,^ ^32^ AM colonization may then shift from being beneficial to neutral or metabolically costly. Colonization percentage alone cannot predict tolerance as highly colonized roots may have poorly functioning arbuscules if C supply, lipid transfer or nutrient exchange is hampered. Functional evaluation should therefore combine photosynthetic stability, host C expenditure, nutrient return, arbuscule activity, fungal identity, wheat genotype, and growth or yield responses.^19^ ^,^ ^32^

### 6.3. Osmoprotectants and metabolic signaling networks

Osmoprotectants contribute to wheat adaptation to salinity. They maintain hydration, protect macromolecules, stabilize membranes, and support metabolism under low external water potential. Osmotic adjustment is often associated with the accumulation of proline, soluble sugars, glycine betaine, amino acids, sugar alcohols, and organic acids.^3^ ^,^ ^8^ ^,^ ^127^ However, these compounds are not only passive solutes; they also act as metabolic signals linking C metabolism, N assimilation, ROS regulation, hormone responses, and ion homeostasis.^6^ ^,^ ^128^

P5CS and P5CR are the main regulators of proline biosynthesis, whereas proline catabolism is regulated by ProDH.^129^ ^,^ ^130^ Glycine betaine accumulation involves BADH and other choline-oxidation enzymes.^117^ Soluble sugars are controlled by sucrose synthases, invertases, trehalose-6-phosphate signaling and sugar transporters, such as SWEET and SUT/SUC families. SWEET and SUT/SUC transporters are particularly important because sugar transport links C allocation, stress signaling, and source-sink coordination under stress.^131^ ^,^ ^132^ Organic acids such as malate and citrate play a role in pH regulation, nutrient mobilization, rhizosphere interactions, and C flux in the tricarboxylic acid cycle Phenolics and flavonoids provide regulatory value as signaling molecules, antioxidants, and mediators of root-microbe communication.^6^ ^,^ ^133^

AM symbiosis modifies osmoprotectant and metabolite networks through better nutrient supply, water relations, and decreased ionic and oxidative stress.^17^ ^,^ ^45^ Improved N nutrition supports proline and amino acid metabolism; S contributes to glutathione synthesis; and P sustains ATP-dependent ion transport and metabolic recovery. Effective osmoprotection therefore depends on coordinated adjustment of proline, sugars, organic acids, glutathione, phenolics, and other membrane-protective metabolites rather than accumulation of a single compound.^17^ ^,^ ^127^ Direct wheat studies confirm changes in several of these metabolites under AM colonization and salinity, but the relationship between individual pathways is still not well established.

High osmolyte accumulation may represent an adaptive response that supports osmotic adjustment, but it may also reflect the severity of stress or impaired growth. Its significance should therefore be interpreted together with indicators such as lipid peroxidation, sugar metabolism, glutathione status, ion balance, and plant growth to distinguish effective metabolic adjustment from stress-associated solute accumulation.

### 6.4. Redox-metabolic integration and symbiotic function

Redox and metabolic signals feed back into root growth and fungal accommodation. ROS are involved in defense and cell-wall remodeling, sugars regulate C exchange between the host and fungus, osmolytes maintain hydration, and antioxidants prevent damage to membranes involved in ion and water transport.^12^ ^,^ ^55^ Their effects are therefore interdependent rather than additive.

AM symbiosis supports a balanced adaptive state by coordinating ROS, ABA, auxin, ethylene, jasmonate, strigolactone signaling, C allocation, and osmoprotectant metabolism. Excessive oxidative and defense signaling restricts root growth and fungal accommodation, whereas insufficient signaling weakens adaptation. AM fungi can help maintain an intermediate state in which roots retain hydraulic function, growth, and arbuscule activity while limiting cellular injury.^32^ ^,^ ^55^ Direct wheat evidence supports the associated improvements in water relations, redox status, and growth, while the precise signaling thresholds that define this state remain to be resolved.

This integration is especially important at the arbuscular interface. Controlled ROS production, membrane trafficking, lipid metabolism, ATP supply, sugar transport, and C transfer are essential for the formation, activity and turnover of arbuscules.^12^ ^,^ ^134^ Arbuscules activity can be impaired by severe salinity, oxidative stress, C limitation or defective lipid transfer, which leads to reduction of symbiotic benefit.^24^ ^,^ ^32^ Conversely, coordinated redox status, nutrient exchange, and C allocation allow functional arbuscules to support ion regulation, antioxidant metabolism, osmotic adjustment, and root resilience.

Overall, AM-mediated redox-metabolic regulation operates across the whole root and the arbuscular interface. Direct wheat evidence supports coordinated improvements in oxidative status, osmolyte metabolism, membrane protection, and growth, whereas the spatial control of ROS and the regulation of sugar and lipid transfer remain based largely on model-system mechanisms. This distinction is essential for explaining why AM symbiosis benefits vary among wheat genotypes, fungal isolates, salinity levels, and developmental stages.

## 7. Hormonal integration of wheat salinity responses and AM symbiosis

Wheat integrates salinity perception, root development, defense activation, ion homeostasis, and symbiotic accommodation through interacting plant hormones.^8^ ^,^ ^25^ Arbuscular mycorrhizal fungi can affect these processes by altering hormone biosynthesis, transport, perception, sensitivity, and downstream transcriptional responses.^31^ ^,^ ^56^ AM symbiosis should therefore be viewed as a modulator of hormonal balance rather than a process that uniformly increases or suppresses individual hormones. Direct evidence in wheat supports hormone-associated changes in root development, stress physiology, and gene expression, whereas detailed links between hormone signaling and fungal accommodation have been resolved primarily in AM model plants and other crop systems.

### 7.1. Hormonal crosstalk among ABA, auxin, ethylene, jasmonate, salicylic acid, and strigolactones

**Figure 3.**
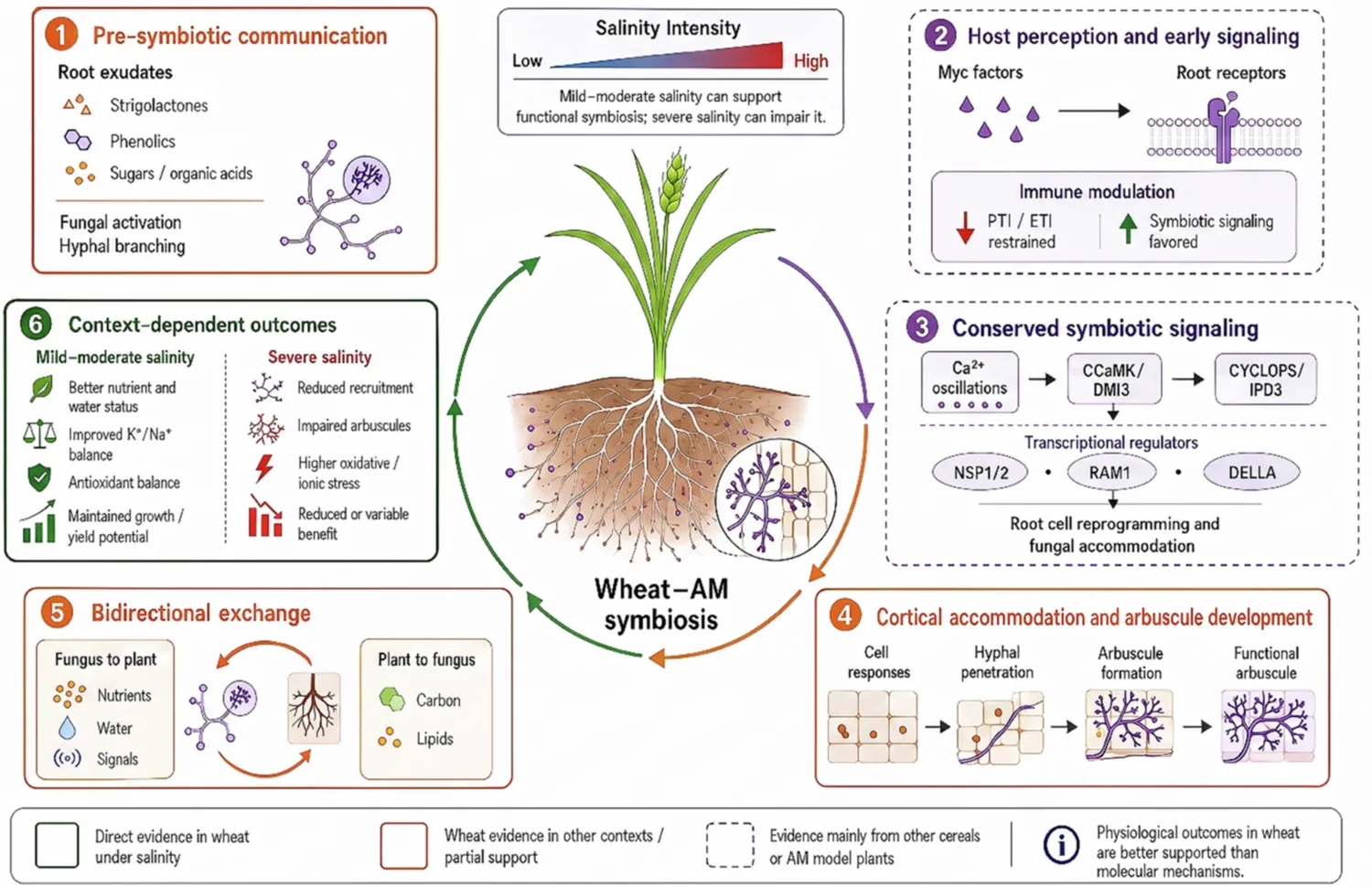
Context-dependent functioning of wheat-AM symbiosis across salinity intensity. The schematic illustrates how osmotic stress, Na⁺ and Cl^−^ imbalance, and ROS signals converge with Ca^2+^ dynamics, hormonal crosstalk, and transcriptional regulation in AM-colonized roots. These interacting pathways coordinate ion homeostasis, aquaporin-mediated water transport, antioxidant and osmoprotectant responses, nutrient exchange, and arbuscule function, thereby supporting membrane stability, root performance, and salinity adaptation. Physiological outcomes are directly supported in wheat, whereas some detailed signaling connections are inferred from other cereals and AM model plants.

Salinity reshapes hormone biosynthesis, transport, perception, and tissue sensitivity, while AM colonization adds the requirement to balance stress protection with fungal accommodation.^13^ ^,^ ^31^ The resulting response depends on the timing, concentration, and spatial distribution of multiple hormones rather than on the action of any single pathway. ABA and strigolactones connect osmotic regulation, nutrient status, and symbiotic development.^135^ ^,^ ^136^ ABA supports stomatal control, osmotic adjustment, and stress-responsive transcription, whereas strigolactone production, particularly under P limitation, promotes presymbiotic activation, and hyphal growth of AM fungi (Figure 3).^137^ However, the coordination of ABA and strigolactone pathways in salt-stressed wheat remains less well characterized than their individual physiological functions.

Root plasticity and defense are influenced by interacting auxin and ethylene pathways together with ABA, jasmonate, salicylic acid, strigolactones, and other developmental regulators. Auxin is necessary for exploratory root development and accommodation of arbuscules; perception of auxin is required for proper arbuscule formation and symbiotic development.^57^ ^,^ ^58^ Ethylene can support adaptive signaling at controlled levels but restrict root growth and fungal colonization when strongly or persistently activated.^31^ ^,^ ^138^ Jasmonic acid and salicylic acid further regulate immune tone and defense-symbiosis balance.^93^ ^,^ ^139^ The relative contribution of each pathway varies with wheat genotype, fungal identity, salinity intensity, nutrient status, and colonization stage.

### 7.2. Spatial, temporal, and threshold-dependent hormone regulation

AM fungi affect hormone networks on three interlinked levels: synthesis, perception, and threshold of response. The direction of these changes is not uniform, because hormone profiles depend on plant species, fungal isolate, tissue, developmental stage, nutrient availability, and stress severity.^31^ ^,^ ^56^ Strigolactone production is tightly linked with P status and AM fungal growth; P limitation increases exudation to stimulate AM fungal spore germination and hyphal branching. Under salinity, patterns of exudation are determined by salt stress, nutrient limitation, C availability, and root metabolism.^43^ ^,^ ^140^ Once AM nutrient exchange is established, improved P can feedback to fine-tune strigolactone biosynthesis. Strigolactone regulation therefore links AM fungal growth with the nutritional return and C cost of the established symbiosis.

Hormonal outcomes also depend on sensitivity, duration, and tissue context. Moderate ABA signaling can support water conservation, whereas sustained activation restricts growth; controlled ethylene and defense signaling may aid acclimation, whereas stronger responses inhibit fungal accommodation.^31^ ^,^ ^56^ Similar concentration- and stage-dependent effects apply to auxin, jasmonate, and salicylate. These threshold-dependent effects help explain why AM symbiosis benefits are highly context-dependent. Under mild to moderate salinity, hormone adjustment can support ABA-mediated water regulation, auxin-dependent root plasticity, controlled ethylene and defense signaling, and strigolactone-mediated fungal communication. Under severe or prolonged salinity, excessive ABA, ethylene, ROS, and defense signaling can overwhelm this balance, restrict host growth and fungal accommodation, and reduce the net symbiotic benefit (Figure 3).^13^ ^,^ ^137^

Most studies of AM-colonized wheat under salinity have evaluated downstream physiological or transcriptional responses rather than simultaneous hormone concentrations, sensitivity, and signaling activity. Consequently, the available evidence is consistent with hormonal coordination, but direct evidence for an integrated, tissue-specific hormonal response in wheat remains limited, and the exact tissue-specific thresholds that determine beneficial versus ineffective symbiosis remain unresolved.

### 7.3. Linking hormone signaling with transcriptional networks and the stress-symbiosis balance

Hormones influence AM-mediated salt tolerance via transcriptional and post-transcriptional networks. ABA-responsive ABF/AREB and bZIP transcription factors activate genes for osmotic adjustment, aquaporins, antioxidants, and ion transport.^141^ ^,^ ^142^ Root development and cortical cell programs required for arbuscule accommodation are regulated by auxin responsive ARFs.^57^ ^,^ ^58^ Ethylene-responsive AP2/ERFs integrate growth restriction, defense and ROS regulation. JA- and SA-related MYC2, JAZ, NPR1, TGA, and WRKY pathways control immune responses and defense balance.^93^ ^,^ ^139^ Strigolactone signaling intersects with NSP1, NSP2, MAX2, and GRAS-family regulators connecting nutrient status, root architecture, and hyphal growth of AM fungi.^143^ ^,^ ^144^ Many of these regulatory relationships have been functionally established in model plants, whereas their combined operation in salt-stressed wheat is supported mainly by conserved gene families and transcriptomic patterns.

AM colonization modifies the hormonal inputs reaching these networks and thereby influences the balance between stress protection and symbiotic development. Transcriptomic studies in wheat support coordinated regulation of ion transport, osmolyte metabolism, antioxidant defense, nutrient acquisition, and fungal accommodation.^14^ ^,^ ^17^ Conserved symbiosis regulators, including CCaMK/CYCLOPS, RAM1, RAD1, DELLA, and WRI5, provide a mechanistic framework for this coordination, but direct wheat-specific evidence linking individual hormone pathways to these regulators under salinity remains limited. Post-transcriptional mechanisms, including small RNAs and alternative splicing, further refine this balance, especially in polyploid wheat.^34^ ^,^ ^35^ Homoeologue-biased expression and stress-responsive splicing are established in wheat, whereas AM-responsive small-RNA and splicing mechanisms are better documented in other plants. Their contribution to combined AM-salinity responses therefore remains plausible but insufficiently tested.

Overall, hormone signaling connects water regulation, root development, defense tone, ion homeostasis, and fungal accommodation. Direct evidence in wheat supports AM-associated changes in these physiological and transcriptional outcomes, whereas model systems provide the molecular architecture linking individual hormones with common symbiosis regulators. The principal unresolved issue is whether these hormonal changes directly regulate symbiotic performance or arise secondarily from improved nutrition and reduced stress injury.

## 8. Future Research Priorities

Despite significant advances in understanding wheat-arbuscular mycorrhizal interactions under salinity, the central priority is to connect the strong physiological and transcriptomic evidence available in wheat with direct functional validation of the signaling mechanisms characterized mainly in AM model plants. Future studies should distinguish descriptive changes in gene expression, metabolites, or antioxidant activity from causal evidence showing that a specific receptor, transporter, transcription factor, or metabolic pathway controls symbiotic performance under salinity.

The temporal and spatial coordination of Ca^2+^, ROS, and hormone signaling remains particularly poorly resolved.^13^ Real-time Ca^2+^ and ROS imaging should therefore be combined with real-time Ca^2+^ and ROS imaging with tissue-resolved profiling of ABA, auxin, ethylene, jasmonate, salicylic acid, and strigolactones. Mutant analysis, RNA interference, genetic complementation, promoter reporters, and fluorescent protein localization have established the functions of CCaMK/DMI3, CYCLOPS/IPD3, RAM1, STR, STR2, and related regulators in AM model systems.^16^ ^,^ ^61^ Applying comparable approaches in wheat, together with CRISPR/Cas editing and homoeologue-specific analysis, can determine whether these conserved regulators perform comparable functions in salt-stressed wheat.

Although HKT, SOS1, NHX, and aquaporins have been implicated in AM-mediated salt tolerance,^13^ ^,^ ^145^ the mechanistic basis of their regulation under combined stress and symbiosis remains largely unexplored in wheat. Genome editing, fluorescent transporter fusions, and ion-specific probes should be integrated with measurements of Na⁺ and K⁺ fluxes, root hydraulic conductivity, nutrient acquisition, and proton-pump activity to distinguish direct transporter regulation from indirect effects of improved nutrition or root growth. Parallel hormone profiling and genetic or pharmacological manipulation would similarly clarify whether hormonal changes drive symbiotic performance or arise secondarily from stress alleviation.

ROS and metabolite networks are central to coordinating stress tolerance, ion transport, osmolyte accumulation, and C allocation.^17^ ^,^ ^32^ Future research should distinguish signaling ROS from oxidative damage through spatial ROS imaging, redox-state measurements, lipid-peroxidation assays, and stage-specific sampling. Metabolomics and stable-isotope tracing can clarify how AM fungi modify osmoprotectant metabolism and reciprocal C, N, and P exchange under salinity. Particular attention should be given to arbuscule functionality because colonization percentage alone does not establish effective symbiosis. Arbuscule abundance, viability, nutrient-transfer activity, fungal C demand, and host growth or yield should be assessed together to determine whether the nutritional return compensates for the plant’s C investment.

A second priority is predictive matching of wheat genotypes with AM fungal isolates. Symbiotic outcomes depend on genotype, fungal identity, salinity intensity, soil properties, and nutrient availability; a single isolate or consortium is therefore unlikely to perform consistently across environments. Systematic screening of diverse wheat-fungus combinations, supported by high-throughput phenotyping and genomic or transcriptomic analysis, could identify markers associated with responsiveness to AM fungal inoculation and salt tolerance. Multi-omics integration can strengthen this predictive framework. Few studies have combined transcriptomics, proteomics, metabolomics, ionomics, and microbiome data to identify regulatory hubs or biomarkers of successful interactions between wheat and AM fungi.^17^ ^,^ ^146^ Developing computational frameworks that combine multi-layer datasets with machine learning or network Network modeling and machine-learning approaches may help prioritize candidate pathways, but predictions must be tested through targeted genetic manipulation, controlled inoculation, and time-resolved phenotyping under defined salinity regimes.

Finally, controlled-environment findings must be translated into field-relevant contexts. Multi-location and multi-season trials across contrasting soil salinity, texture, moisture, and nutrient regimes should compare selected wheat-fungus combinations, inoculation methods, and interactions with irrigation and fertilization.^8^ These trials should assess colonization persistence, arbuscule functionality, nutrient- and water-use efficiency, grain yield, and yield stability. Future research should therefore progress from descriptive physiology and omics to causal validation, genotype-fungus matching, and multi-environment field testing. This sequence will connect mechanisms established in AM model plants with wheat-specific evidence and support breeding, inoculant development, and agronomic deployment in salt-affected environments.

## 9. Conclusion

Current evidence establishes AM symbiosis as an important modifier of wheat responses to salinity. Studies, particularly in durum wheat, consistently report improved nutrient acquisition, K⁺/Na⁺ balance, water relations, membrane stability, antioxidant regulation, osmoprotectant metabolism, and growth. Transcriptomic findings further indicate that AM-colonized plants enter a distinct stress-symbiosis state. However, these physiological and transcriptional outcomes are better established than the molecular pathways proposed to generate them. Mechanisms involving fungal-signal perception, symbiotic Ca^2+^ decoding, GRAS transcription factors, spatial ROS regulation, hormonal thresholds, arbuscule maintenance, and plant-to-fungus lipid transfer have been characterized mainly in AM model plants and other cereals and require direct validation in wheat under salinity.

The symbiosis between wheat and AM fungi is a context-dependent interaction whose outcome depends on reciprocal resource exchange. Wheat genotype, AM fungal identity, salinity severity, nutrient availability, developmental stage, soil conditions, and host C allocation determine whether fungal benefits exceed the metabolic cost of maintaining the symbiosis. Colonization percentage alone is therefore insufficient to demonstrate functional mutualism; arbuscule viability, nutrient-transfer activity, extraradical hyphal function, C investment, and plant growth or yield should be evaluated together.

Future research should progress from physiological and omics-based associations to causal validation and field translation. Wheat-specific gene editing, reporter systems, ion and hormone profiling, redox imaging, isotope tracing, and quantitative arbuscule phenotyping can clarify how conserved symbiotic pathways interact with salinity responses. Coupling these approaches with genotype-fungus screening and multi-environment field trials will support the integration of responsiveness to AM fungal inoculation with established salinity breeding and crop-management strategies for productive wheat cultivation in salt-affected soils.

## Supplementary Material

Supplementary Material Supplementary_table_1_FINAL.docx

## Data Availability

No new data was generated for this study.
