# Supplementary material for: Responses of arbuscular mycorrhizal wheat to salinity: from symbiotic signaling to stress adaptation
Source: Plant Signal Behav. 2026 Sep 23;21(1):2733240. doi: 10.1080/15592324.2026.2733240 (PMC13613886; doi:10.1080/15592324.2026.2733240)
Supplement: Supplementary file 1 [file KPSB_A_2733240_SM2963.docx]

**Table S**1. Experimental characteristics and principal findings of primary studies directly examining arbuscular mycorrhizal wheat under salinity.

| **Wheat genotype** | **AM fungal species/isolate** | **Salinity treatment and growth conditions** | **Variables evaluated** | **Major findings** | **Reference** |
| --- | --- | --- | --- | --- | --- |
| *Triticum aestivum* cvs. Roshan and Kavir; mutated Tabasi line | *Glomus etunicatum*, *G. mosseae* and *G. intraradices*; separate inocula | Field experiment; soil EC 7.41 dS m⁻¹; irrigation-water EC 13.87 dS m⁻¹ | Root colonization, biomass, yield components, nutrient uptake, shoot Na⁺ and Cl⁻ | Responses depended on genotype and fungal species. The Tabasi–*G. etunicatum* combination produced the greatest biomass and grain-yield increases and lower shoot Na⁺ and Cl⁻ | ^1^ |
| *T. aestivum* cvs. Sakha 8 and Giza 167 | Mixed *Glomus* spp. inoculum | Greenhouse pot experiments over two seasons; EC 0.15, 3.13, 6.25 and 9.38 dS m⁻¹ | Root colonization, yield, mineral nutrients, soluble sugars, amino acids, proline, POD and CAT | Inoculation increased nutrient uptake, osmolyte accumulation, antioxidant activity and yield and reduced Na⁺ accumulation; responses were stronger in Sakha 8 | ^2^ |
| Field: Roshan, Kavir and Tabasi; greenhouse: Chamran and Line 9 | *G. mosseae*, *G. etunicatum* and *G. intraradices*; separate and mixed inocula | Field soil EC 7.41 dS m⁻¹ and irrigation-water EC 13.87 dS m⁻¹; greenhouse EC 3.9, 7.85 and 12.1 dS m⁻¹ | Root colonization, biomass, macro- and micronutrient uptake, Na⁺ and Cl⁻ | All fungal treatments improved nutrient acquisition; the mixed inoculum produced synergistic responses and restricted Na⁺ accumulation more strongly than Cl⁻ accumulation | ^3^ |
| *T. aestivum* cv. Henta | *G. mosseae*, *G. deserticola* and *Gigaspora gergaria* | Greenhouse pots containing naturally saline sandy soil; measurements after 8 and 12 weeks | Growth, pigments, mineral nutrients, phosphatases, proline, soluble protein, colonization and fungal metabolic activity | All fungi improved growth and mineral nutrition and reduced shoot Na⁺; *G. mosseae* generally produced the strongest response | ^4^ |
| *T. aestivum* cvs. Sids 1 and Giza 168 | Mixed *Glomus* spp. inoculum | Greenhouse pot experiments over two seasons; EC 0.1, 4.7 and 9.4 dS m⁻¹ | Colonization, productivity, RWC, MSI, photochemistry, gas exchange, C and N metabolism, osmolytes, H₂O₂ and MDA | Inoculation improved photosynthesis, water status, C–N metabolism and productivity and reduced oxidative injury; effects were more pronounced in Sids 1 | ^5^ |
| *T. aestivum* cv. Hereford | *Rhizophagus irregularis* | Controlled glasshouses at 400 or 700 µmol CO₂ mol⁻¹; EC 0, 9.5 and 19.0 dS m⁻¹; 10 weeks | Colonization, biomass, stomatal traits, gas exchange, leaf water potential, NUE, soluble sugars and K⁺/Na⁺ ratio | The symbiosis increased stomatal conductance, NUE, soluble sugars and K⁺/Na⁺ balance and supported biomass accumulation, particularly under elevated CO₂ and salinity | ^6^ |
| Durum wheat, cv. Anco Marzio | *R. irregularis* and *Funneliformis mosseae*; mixed inoculum | Outdoor pots; 0 or 10 g NaCl L⁻¹ applied progressively; final soil EC approximately 13 dS m⁻¹; harvest at 45 days | Colonization, biomass, SPAD, MSI, N uptake and targeted expression of N-transporter, aquaporin and stress-responsive genes | Mycorrhizal plants maintained greater biomass, N uptake and MSI; NRT1.1 was upregulated, whereas induction of several aquaporin and stress-responsive genes was attenuated | ^7^ † |
| *T. aestivum* cv. Hereford | *R. irregularis* | Controlled pot experiment; ambient or 700 ppm CO₂; 0, 1 or 2 g NaCl kg⁻¹ dry soil | Colonization, shoot biomass, C, N, K, Ca, Mg and Na accumulation, soluble sugars and glucose | The symbiosis increased biomass, carbohydrate assimilation and nutrient accumulation; salinity reduced root colonization, whereas elevated CO₂ increased it | ^8^ |
| Spring wheat, cv. 1110 (Kloka WM1353) | Five-isolate consortium: *R. irregularis* including BEG140, *F. mosseae* BEG95, *F. geosporum* and *Claroideoglomus claroideum* | Greenhouse experiment; 200 mM NaCl imposed before anthesis, after anthesis or at both stages; five replicates | Colonization, growth, yield components, chlorophyll, gas exchange and plant C and N | Inoculated plants showed greater net photosynthesis and stomatal conductance; grain yield was maintained when salinity occurred at both developmental stages | ^9^ |
| *T. aestivum* cv. AAS-2011 | Commercial *Glomus* spp. inoculum | Greenhouse pots; 0 or 200 mM NaCl applied progressively; 12 weeks | Growth, yield, pigments, fluorescence, RWC, electrolyte leakage, H₂O₂, MDA, antioxidants, nutrients and Na⁺/K⁺ balance | Inoculation improved growth, yield, pigments, water status, antioxidant capacity and nutrient uptake and reduced Na⁺ accumulation, membrane leakage and oxidative injury | ^10^ |
| Durum wheat, cv. Anco Marzio | *R. irregularis* and *F. mosseae*; mixed inoculum | Outdoor pots; 0 or 10 g NaCl L⁻¹ applied progressively; final soil EC approximately 13 dS m⁻¹; harvest at 45 days | Colonization, biomass, N uptake, MSI, leaf RNA sequencing and qPCR validation | Mycorrhizal plants maintained greater biomass, N uptake and MSI; 563 differentially expressed genes indicated regulation of stress, metabolism, RNA processing, vesicle trafficking and cell-wall pathways | ^11^ † |

**Notes:** Fungal names are presented according to the nomenclature used in the original publications. Where an article reported an inoculum only at genus or consortium level, that taxonomic resolution was retained rather than assigning an unreported isolate. **†** Studies containing molecular-level analyses. The other nine studies primarily evaluated physiological, biochemical, nutritional, photosynthetic, agronomic or yield-related responses. Abbreviations: AM, arbuscular mycorrhizal; CAT, catalase; EC, electrical conductivity; MDA, malondialdehyde; MSI, membrane stability index; NUE, nitrogen-use efficiency; POD, peroxidase; RWC, relative water content.

**References**

1.	Daei G, Ardekani MR, Rejali F, Teimuri S, Miransari M. Alleviation of salinity stress on wheat yield, yield components, and nutrient uptake using arbuscular mycorrhizal fungi under field conditions. J Plant Physiol 2009; 166:617–25.

2.	Talaat NB, Shawky BT. Influence of arbuscular mycorrhizae on yield, nutrients, organic solutes, and antioxidant enzymes of two wheat cultivars under salt stress. J Plant Nutr Soil Sci 2011; 174:283–91.

3.	Mardukhi B, Rejali F, Daei G, Ardakani MR, Malakouti MJ, Miransari M. Arbuscular mycorrhizas enhance nutrient uptake in different wheat genotypes at high salinity levels under field and greenhouse conditions. Comptes Rendus Biol 2011; 334:564–71.

4.	Abdel-Fattah GM, Asrar A-WA. Arbuscular mycorrhizal fungal application to improve growth and tolerance of wheat (Triticum aestivum L.) plants grown in saline soil. Acta Physiol Plant 2012; 34:267–77.

5.	Talaat NB, Shawky BT. Protective effects of arbuscular mycorrhizal fungi on wheat (Triticum aestivum L.) plants exposed to salinity. Environ Exp Bot 2014; 98:20–31.

6.	Zhu X, Song F, Liu S, Liu F. Role of arbuscular mycorrhiza in alleviating salinity stress in wheat (Triticum aestivum L.) grown under ambient and elevated CO2. J Agron Crop Sci 2016; 202:486–96.

7.	Fileccia V, Ruisi P, Ingraffia R, Giambalvo D, Frenda AS, Martinelli F. Arbuscular mycorrhizal symbiosis mitigates the negative effects of salinity on durum wheat. PLoS One 2017; 12:e0184158.

8.	Zhu X, Song F, Liu S, Liu F, Li X. Arbuscular mycorrhiza enhances nutrient accumulation in wheat exposed to elevated CO2 and soil salinity. J Plant Nutr Soil Sci 2018; 181:836–46.

9.	Eroğlu ÇG, Cabral C, Ravnskov S, Bak Topbjerg H, Wollenweber B. Arbuscular mycorrhiza influences carbon‐use efficiency and grain yield of wheat grown under pre‐and post‐anthesis salinity stress. Plant Biol 2020; 22:863–71.

10.	Huang S, Gill S, Ramzan M, Ahmad MZ, Danish S, Huang P, Al Obaid S, Alharbi SA. Uncovering the impact of AM fungi on wheat nutrient uptake, ion homeostasis, oxidative stress, and antioxidant defense under salinity stress. Sci Rep 2023; 13:8249.

11.	Puccio G, Ingraffia R, Mercati F, Amato G, Giambalvo D, Martinelli F, Sunseri F, Frenda AS. Transcriptome changes induced by Arbuscular mycorrhizal symbiosis in leaves of durum wheat (Triticum durum Desf.) promote higher salt tolerance. Sci Rep 2023; 13:116.
